# A common variant in 11q23.3 associated with hyperlipidemia is mediated by the binding and regulation of GATA4

**DOI:** 10.1038/s41525-021-00279-5

**Published:** 2022-01-19

**Authors:** Wen-Cheng Chou, Wei-Ting Chen, Chen-Yang Shen

**Affiliations:** 1 Institute of Biomedical Sciences, Academia Sinica, Taipei, Taiwan; 2 College of Public Health, China Medical University, Taichung, Taiwan

**Keywords:** Epidemiology, Genetic association study, Metabolic syndrome, Predictive markers, Gene expression analysis

## Abstract

Large-scale genome-wide associations comprising multiple studies have identified hundreds of genetic loci commonly associated with hyperlipidemia-related phenotypes. However, single large cohort remains necessary in aiming to investigate ethnicity-specific genetic risks and mechanical insights. A community-based cohort comprising 23,988 samples that included both genotype and biochemical information was assembled for the genome-wide association analysis (GWAS) of hyperlipidemia. The analysis identified fifty genetic variants (*P* < 5 × 10^−8^) on five different chromosomes, and a subsequent validation analysis confirmed the significance of the lead variants. Integrated analysis combined with cell-based experiments of the most statistically significant locus in 11q23.3 revealed rs651821 (*P* = 4.52 × 10^−76^) as the functional variant. We showed transcription factor GATA4 preferentially binds the T allele of rs651821, the protective allele for hyperlipidemia, which promoted *APOA5* expression in liver cells and individuals with the TT genotype of rs651821. As GATA4-APOA5 axis maintains triglyceride homeostasis, GATA4 activation by phenylephrine implies synergism for lowering triglyceride levels in hyperlipidemia patients. Our study demonstrates that rs651821 mediates *APOA5* activation via allele-specific regulation by GATA4. We suggest elevating GATA4 activity could provide a therapeutic potential for treating the development of hyperlipidemia.

## Introduction

Dysregulation of plasma lipid levels contributes substantially to the development of several metabolic disorders, including cardiovascular diseases and type 2 diabetes mellitus^1–4^. Hyperlipidemia is primarily diagnosed by detecting elevated levels of triglycerides (TG) and/or total cholesterol (TC) in plasma samples^5^. Hyperlipidemia is a complex polygenic trait, and TG and TC levels in plasma are considered to be highly heritable^6,7^. In recent decades, genome-wide association studies (GWAS) have identified hundreds of loci that are associated with altered levels of TG, TC, low-density lipoprotein cholesterol (LDL-C), or high-density lipoprotein cholesterol (HDL-C)^7,8^. Most of these loci were initially identified in studies of populations of European ancestry (EUR) and later validated and confirmed in studies of East Asian populations (EAS)^9^. Genetic variants found with top-ranked significance are quite consistent between analyses of European and East Asian ancestors^9^. These include TC-associated loci in genes encoding the apolipoproteins *APOE/C1/C2* (rs7412) and *SORT1* (rs599839/rs629301) and TG-associated loci in *APOA1/C3/A4/A5* (rs662799/rs964184) and *GCKR* (rs1260326) genes. However, comparisons of loci among different ethnic groups have often shown that the magnitude of effect of each genetic variation can differ between populations. For example, in a multicenter study regarding EAS, researchers identified four new variants that were significantly (*P* < 5 × 10^−8^) associated with the levels of TG (rs10886863) and HDL-C (rs10743940, rs10504476 and rs10144765)^9^, yet none of these variants was significant (*P* > 0.13) for EUR^8^. These findings underscore the importance of considering ethnicity and population aspects when evaluating the significance of loci associated with the same metabolic traits.

Given that 88–94% of genetic variants identified from GWAS reside in non-coding regions, any effects on gene expression and disease phenotypes are expected to be mediated via regulation of promoters or enhancers^10,11^. Equally important, a growing body of evidence has shown that the lead single nucleotide polymorphisms (SNPs) identified by GWAS are rarely the causal variants that determine the traits^12,13^. Consequently, the identification of true causal variants is critical for translating GWAS findings to disease prevention/intervention as well as for understanding the mechanism/etiology of diseases. Nevertheless, only a small portion of variants, i.e., among the >100 hyperlipidemia-associated loci identified by GWAS, have been elucidated with respect to their impact on the regulation of the lipid metabolism factors *SORT1* and *GCKR* as well as *VLDLR* and *ELOVL2*^14–17^. This result clearly shows that the biological/pathological contributions of the majority of GWAS-significant variants remain to be resolved.

In the present study, we analyzed hyperlipidemia-related genetic risk loci based on study participants from the Taiwan Biobank (TWB), which is the single largest cohort comprised of 23,988 individuals in Taiwan^18^. To define the pathological contribution of genetic variants, particular attention was given to the top-ranked locus 11q23.3, which revealed variant rs651821 as the causal variant affecting TG level, rather than variant rs662799 that has been repeatedly reported in several studies^3,8,9^. Additional functional experiments revealed that the transcription factor GATA4 binds specifically to the protective allele of rs651821 to modulate *APOA5* expression and TG level. Consistently, similar result was observed through our in vivo analysis of clinical samples. We conclude that a genetic and molecular linkage exists between GATA4 and APOA5 via rs651821, which contributes to the maintenance of TG homeostasis.

## Results

### Identification of hyperlipidemia-associated genetic loci

**Fig. 1:**
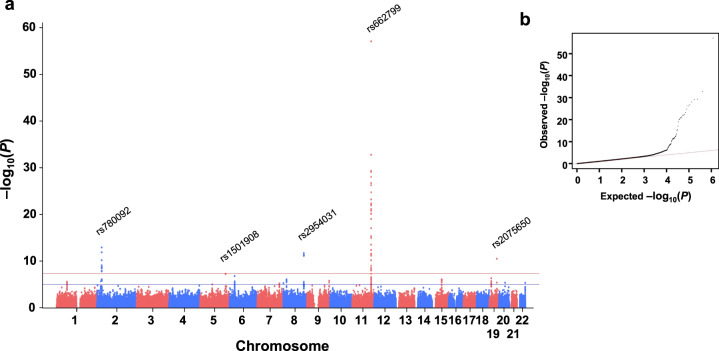
Genome-wide analysis for hyperlipidemia. **a** Manhattan plot of results for the association between SNPs and hyperlipidemia risk. **b** Q-Q plot. The two horizontal lines in the Manhattan plot represent *P* = 1 × 10^−5^ (lower) and 5 × 10^−8^ (upper). The estimated inflation factor (λ) was 1.019 for the GWAS.

At the discovery stage of the GWAS analysis, 50 statistically significant SNPs (*P* < 5 × 10^−8^) were identified on chromosomes 2p23.3, 5q33.3, 8q24.13, 11q23.3, and 19q13.32 (see Fig. 1 for Manhattan and Q-Q plots). Notably, all SNPs in each of these five loci were clustered, suggesting they do not represent random events. After confirming the independence of these SNPs, we chose the top SNP in each of the five loci: rs780092 (2p23.3), rs1501908 (5q33.3), rs2954031 (8q24.13), rs662799 (11q23.3), and rs2075650 (19q13.32). The validation/replication stage of the GWAS, which was based on an independent set of study participants, confirmed the importance of each SNP, and all SNPs remained significantly associated with hyperlipidemia (*P* < 0.001, Table 1, and Supplementary Table 1). The results combined from the two stages confirmed that all five SNPs identified by the GWAS consistently reached statistical significance (5 × 10^−8^, Table 1) We checked these five SNPs in the GWAS catalog database (https://www.ebi.ac.uk/gwas/) and confirmed that these five known loci are bona fide important in determining hyperlipidemia not only in the Taiwanese population but also in other EAS (Supplementary Table 2).

**Table 1:** Top five lead variants identified in the GWAS.

				Discovery (*N* = 9714)			Validation (*N* = 2662)		Combination	
SNP	Loci	Nearest gene	Allele	MAF	OR (95% CI)	*P* value	OR (95% CI)	*P* value	OR (95% CI)	*P* value
rs662799	11q23.3	APOA5	A/G	0.28	1.80 (1.68–1.94)	5.90 × 10^–58^	1.84 (1.60–2.11)	7.23 × 10^–18^	1.79 (1.68–1.91)	1.22 × 10^–76^
rs780092	2p23.3	GCKR	A/G	0.36	0.77 (0.72–0.83)	6.60 × 10^–14^	0.70 (0.61–0.80)	1.51 × 10^–7^	0.77 (0.73–0.82)	7.06 × 10^–18^
rs2954031	8q24.13	TRIB1	T/G	0.47	1.26 (1.18–1.34)	1.52 × 10^–12^	1.21 (1.07–1.37)	2.62 × 10^–3^	1.23 (1.17–1.30)	9.12 × 10^–14^
rs2075650	19q13.32	TOMM40	A/G	0.082	1.46 (1.31–1.64)	3.25 × 10^–11^	1.49 (1.20–1.85)	3.29 × 10^–4^	1.43 (1.30–1.58)	2.53 × 10^–13^
rs1501908	5q33.3	TIMD4	C/G	0.27	0.81 (0.76–0.88)	4.80 × 10^–8^	0.80 (0.69–0.92)	1.93 × 10^–3^	0.84 (0.79–0.89)	2.27 × 10^–8^
Imputed										
rs651821	11q23.3	APOA5	T/C	0.28	1.80 (1.68–1.94)	4.75 × 10^–57^	1.84 (1.60–2.11)	9.46 × 10^–18^	1.79 (1.68–1.91)	4.52 × 10^–76^

*SNP* single-nucleotide polymorphism, *MAF* minor allele frequency, *OR* odds ratio, *CI* confidence interval.

### Variant rs651821 is the most likely functional variant at locus 11q23.3

**Fig. 2:**
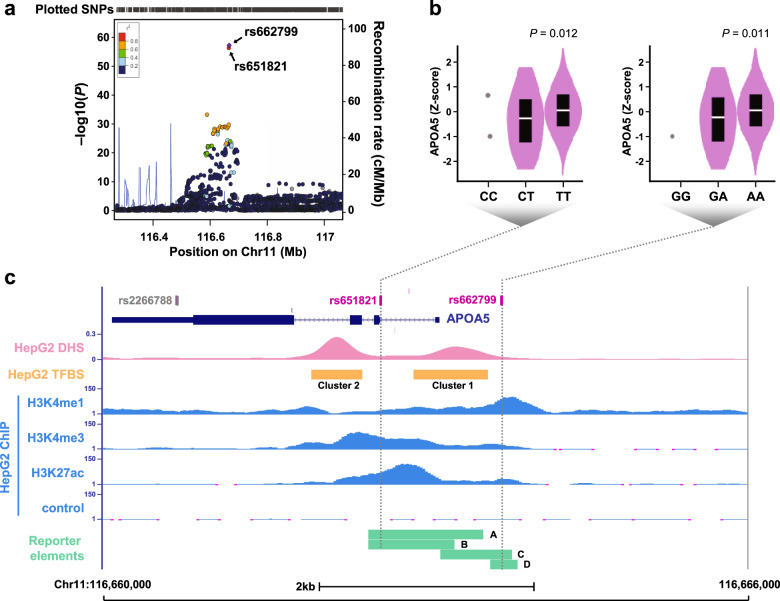
Integrated analyses to prioritize the causal variant at locus 11q23.3. **a** Regional association plot for hyperlipidemia in association with the top SNP rs662799 (purple diamond). The other GWAS-detected/imputed SNPs are displayed by color showing their degrees of linkage (*r*^2^) with rs662799. **b** The eQTL correlation between *APOA5* expression and the rs651821 genotype (left) or rs662799 (right) in liver tissues, for which the plot and *P* values were queried from the GTEx Portal. **c** Visualization of epigenetic regulation in HepG2 cells including histone modifications, DNase hypersensitivity region (DHS), and transcription factor binding sites (TFBS) upstream of *APOA5* (UCSC Genome Browser http://genome.ucsc.edu/cgi-bin/hgPublicSessions, session name: Liver_APOA5p). The locations of the two candidate SNPs (rs651821 and rs662799) as well as the previously described rs2266788 are indicated. DNA fragments used for reporter analysis in the present study are indicated (elements A to D).

Admittedly, the five loci identified by the GWAS are not novel, and rs662799 in 11q23.3 has been identified as the top locus for hyperlipidemia in several studies, including the present one. Variant rs662799 is located upstream of the *APOA5* promoter, and APOA5 is a well-established regulator of blood lipid metabolism^19^. Therefore, we reasoned that *APOA5* expression may be regulated by different alleles of rs662799. In other words, lower *APOA5* expression and higher TG level is associated with the G allele of rs662799 (rs662799-G), whereas higher *APOA5* expression and lower TG level is associated with the rs662799-A. Not surprisingly, however our analysis as well as previous studies excluded the involvement of rs662799 in *APOA5* expression (Supplementary Fig. 1)^20,21^. This implied that the downstream gene expression between the fragments containing the G or A allele of rs662799 is not regulated by transcription factors that are predicted to bind this SNP. Therefore, putative causal SNPs at locus 11q23.3 that affect APOA5 level remained to be identified. Toward this end, we imputed all SNPs within a 2-Mb region centered on rs662799 and conducted the analysis again. Variant rs662799 remained the most significant SNP associated with hyperlipidemia, but another SNP, i.e., rs651821, with a high degree of linkage disequilibrium (LD) with rs662799 (*r*^2^ = 0.99, our cohort), attracted our attention because it was the second most significant variant (Fig. 2a and Table 1). Similar to rs662799, rs651821 has been previously associated with TG level^8^. In addition to *APOA5*, three other apolipoprotein genes, namely *APOA1*, *APOC3* and *APOA4*, are located at locus 11q23.3, but the rs651821-C and rs662799-G were only specifically correlated with lower *APOA5* expression (Fig. 2b and Supplementary Table 3).

Variant rs651821 is located adjacent to the *APOA5* transcription start site. However, no study has explored the mechanism by which rs651821 regulates *APOA5* expression. Functional annotation based on ENCODE revealed that rs651821 colocalized within the overlapping region of H3K4me3 and H3K27ac (Fig. 2c), the histone epigenetic marks suggesting active transcription and an open chromatin region. This information suggested that rs651821 may be a causal variant that affects *APOA5* expression. As such, we prioritized rs651821 over rs662799 and conducted experiments to explore this possibility.

### GATA4 specifically binds the T allele of rs651821

**Fig. 3:**
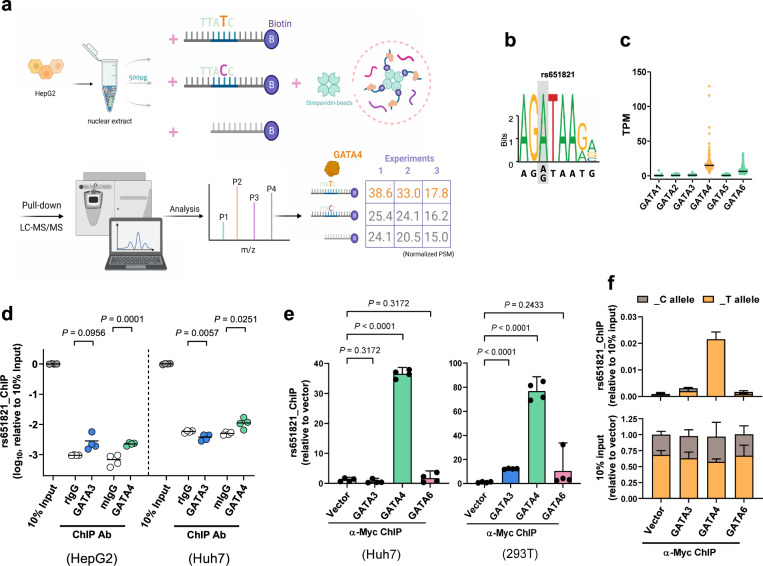
Identification of transcription factors that preferentially bind rs651821. **a** Schematic diagram of the mass spectrometry screen for transcription factors that bind rs651821. Enriched peptides corresponding to GATA4 were normalized as indicated. PSM, peptide spectrum matches. The figure was created with BioRender.com. **b** GATA consensus motif retrieved from the JASPAR 2020 database (http://jaspar.genereg.net/). **c** Distribution and differential expression of individual members of the *GATA* family in GTEx liver tissues. **d** HepG2- and Huh7-cell DNA fragments after ChIP with normal rabbit IgG, normal mouse IgG, anti-GATA3, or anti-GATA4 were quantified by SYBR-based qPCR. **e** Histogram showing the relative rs651821 site enrichment after ChIP with anti-Myc using Huh7 or 293 T cells transfected with pXJ vectors expressing Myc-tagged GATA proteins. **f** DNA fragments before (lower panel) and after (upper panel) ChIP with anti-Myc performed in 293 T cells (**e**) were quantified by TaqMan-based qPCR for rs651821. The enriched specific allele in each group was calculated and normalized by the respective FAM^ΔCt^ or VIC^ΔCt^ of 10% input, and the result is displayed by the addition of 2^–ΔCt^ (FAM + VIC). *P* values and 95% CI (error bars) for the results within the figure are indicated; three independent experiments of *n* = 4 were performed.

*APOA5* is expressed solely in liver tissue (Supplementary Fig. 2), and therefore it was anticipated that experiments carried out with the liver cell line HepG2 (derived from a young hepatoblastoma with high-level *APOA5* expression) would yield physiologically relevant results (Supplementary Fig. 3). However, based on ENCODE, no known transcription factors were located at the regions covering rs651821 in HepG2 cells (Fig. 2c). To address this apparent conundrum, we designed biotinylated oligonucleotide duplex containing the rs651821 site to pulldown interacting proteins from HepG2 nuclear extract. Quantitative mass spectrometry analysis revealed one canonical transcription factor, namely GATA4 that bound rs651821 in a T allele–preferential manner (Fig. 3a). This possibility was supported by the fact that GATA4 binds the consensus nucleotide motif G-A-T-A that resides upstream of many promoters. GATA4, together with the other five GATA proteins, comprises the GATA transcription factor family. Polymorphism of rs651821 occurred exactly at the first A allele that changes the G-A-T-A motif to G-G-T-A, and theoretically, all GATA proteins would preferentially bind to the rs651821-T (Fig. 3b). Among all six GATA genes, only *GATA4* was highly expressed in liver; *GATA3* and *GATA6* were expressed at low levels, and *GATA1*, *GATA2* and *GATA5* were nearly undetectable (Fig. 3c). ChIP analysis of HepG2 and Huh7 (another liver line) cells confirmed GATA4 enrichment at rs651821 (Fig. 3d). Moreover, in Huh7 and 293 T cells overexpressing GATA3, GATA4, or GATA6 (or vector alone, as a control; Supplementary Fig. 4), only GATA4 was substantially bound to chromatin (Fig. 3e). The fact that 293 T cells are heterozygous at rs651821—harboring both the T allele and C allele (Supplementary Fig. 5)—afforded the opportunity to examine allele-specific binding of GATA4. Consistent to our expectation, GATA4 was confirmed preferentially bound to the rs651821-T by the use of ChIP assay followed by TaqMan-based quantification (Fig. 3f). Notably, rs651821 is located within *APOA5*, being at the front of exon 2, but *APOA5* expression still could be regulated by the region containing rs651821, as evidenced by results from experiments with CRISPR activation or interference systems with two different proximal single guide RNAs (sgRNAs) targeting the region adjacent to rs651821 (Supplementary Fig. 6). These findings yielded supportive clues that GATA4 can regulate *APOA5* expression in liver cells by specifically binding the rs651821-T.

### Differential *APOA5* regulation by GATA4 depends on the rs651821 genotype

**Fig. 4:**
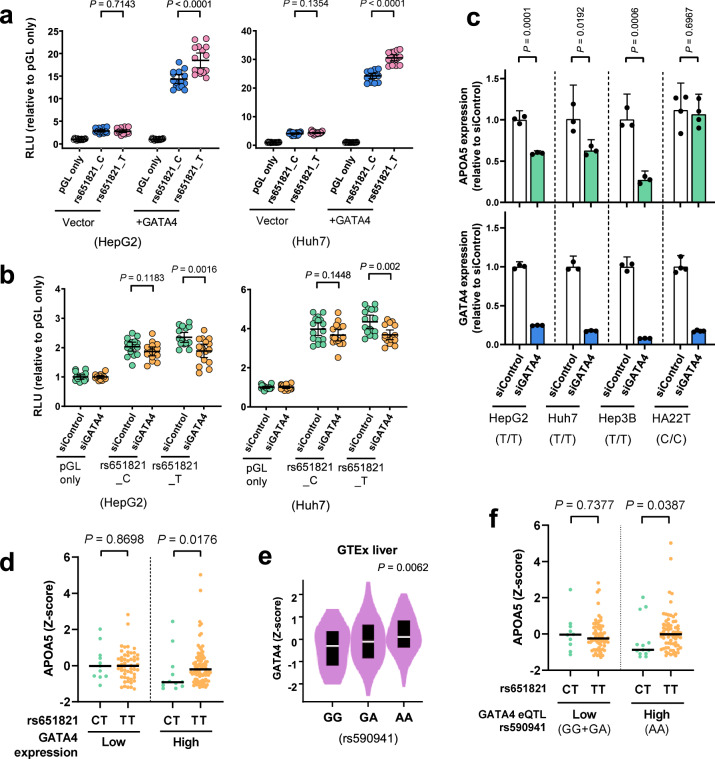
Allelic imbalance in *APOA5* expression is mediated by GATA4. **a** Reporter analysis of DNA fragments containing different rs651821 genotypes (element B) in HepG2 cells (left) or Huh7 cells (right) transfected with empty vector or GATA4-overexpressing vector. RLU relative luminescence units. **b** Reporter analysis of DNA fragments containing different rs651821 genotypes in HepG2 cells (left) or Huh7 cells (right) that had been subjected to knockdown by control siRNA or siRNA targeting *GATA4*. *P* values and 95% CIs for the results are indicated; pools of four independent luciferase experiments of *n* = 4 are shown as dot plots. **c** Histogram showing relative *GATA4* and *APOA5* expression in liver cells transfected with control siRNA or siRNA targeting *GATA4*, as determined by qPCR. The rs651821 genotype for each cell line was examined by Sanger sequencing as indicated below the graphs. At least two independent transfections of *n* = 3 (*n* = 4 in HA22T cells) were performed, and the *P* values and 95% CIs are indicated. **d** *Z* scores for *APOA5* expression sequentially stratified by *GATA4* expression and rs651821 genotypes based on the information retrieved from the GTEx liver dataset. Median values are indicated. Statistical difference between two groups was determined by the Mann–Whitney *U* test. **e** The significant *GATA4* eQTL in liver tissues queried from the GTEx dataset. **f** *Z* scores for *APOA5* expression sequentially stratified by rs590941 and rs651821 genotypes and analyzed as described in (**d**).

We next examined whether rs651821 regulates *APOA5* expression. Liver cell lines were co-transfected with vector pGL4.23 encoding the T or C allele of rs651821 and a vector expressing GATA4, after which luciferase activity was measured. In HepG2 cells, while the DNA fragments containing rs651821 showed reporter activity compared to empty control, no difference was detected between the two C and T alleles in the absence of *GATA4* transfection (Fig. 4a). GATA4 overexpression increased the reporter activity of cells with the rs651821-T (Fig. 4a), and this was also the case for the liver cell lines Huh7 and Huh6 (Fig. 4a and Supplementary Fig. 7a). In contrast, *GATA4* knockdown via a siRNA in these three liver cell lines decreased the reporter activity of the fragment containing the rs651821-T without affecting that of the rs651821-C (Fig. 4b and Supplementary Fig. 7b). Additional experiments with these cell lines carrying the rs651821-TT genotype revealed that *GATA4* knockdown significantly downregulated *APOA5* expression (Fig. 4c). In contrast, *GATA4* knockdown in HA22T/VGH liver cells (carrying the rs651821-CC) did not significantly reduce *APOA5* mRNA level (Fig. 4c). These results were consistent with those of our mass spectrometry and ChIP analyses, indicating that GATA4 regulates *APOA5* expression in a rs651821 T allele–specific manner.

Having demonstrated that GATA4 modulates *APOA5* expression, we next assessed their genetic interaction in population dataset. The result from the GTEx dataset (Fig. 2b) had already demonstrated significantly greater *APOA5* expression for the rs651821-TT group than the rs651821-TC group. In fact, upon higher *GATA4* expression (*n* = 95), *APOA5* expression was higher for the rs651821-TT group (83 of 95) than the rs651821-TC group (12 of 95). However, when *GATA4* was lower expressed (*n* = 65), *APOA5* expression did not differ between those two groups (Fig. 4d), implying the involvement of GATA4 in modulating the *APOA5* eQTL. To further confirm our hypothesis, we searched for a SNP that represented *GATA4* expression in liver tissue, according to GTEx (Supplementary Dataset 1). This revealed that rs590941 is conditionally the most significant *cis*-eQTL for *GATA4* (Fig. 4e). The rs590941-AA genotype is associated with higher cellular level of *GATA4* in liver, implying GATA4 activation. The observed greater *APOA5* expression in the rs651821-TT group (*n* = 64) than in the rs651821-TC group (*n* = 10) remained statistically significant in the samples carrying the rs590941-AA genotype, indicating a genetic contribution from *GATA4* expression (Fig. 4f). However, for the rs590941-GG/GA genotype (*n* = 82), which is associated with lower *GATA4* expression, *APOA5* expression was not significantly higher in the rs651821-TT group (*n* = 69) (Fig. 4f). Notably, *APOA5* expression did not correlate with rs590941 (the *cis*-eQTL for *GATA4*) (Supplementary Fig. 8). Therefore, the discriminate role of rs590941 in modulating the association between rs651821 and *APOA5* expression probably reflects direct regulation by GATA4. In total, the findings suggested a role for rs651821 in modulating *APOA5* expression via allele-specific recruitment of GATA4 to the *APOA5* promoter.

### Elevated GATA4 contributes to TG homeostasis

**Fig. 5:**
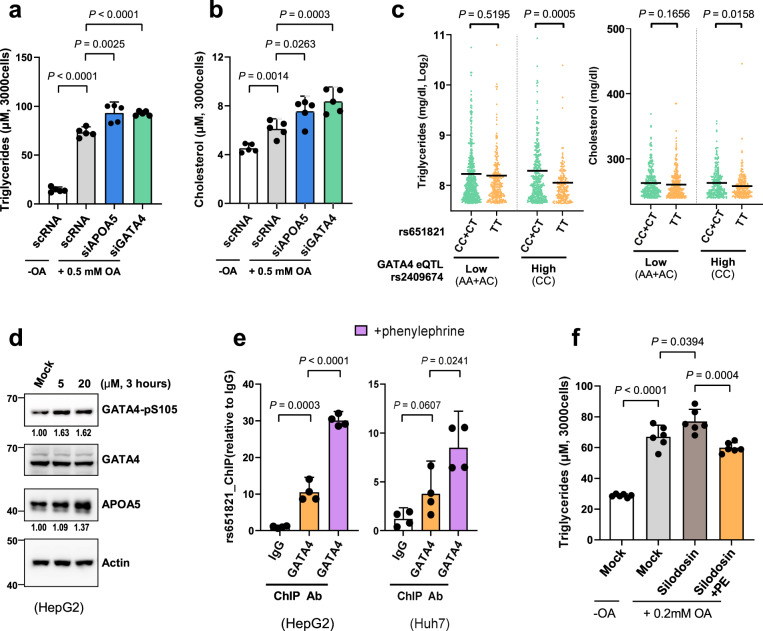
Regulatory role of GATA4 in cellular and plasma TG homeostasis. **a**, **b** Quantification of TG (**a**) or TC (**b**) level in HepG2 cells treated with or without oleic acid (OA). *P* values and 95% CIs are indicated, and independent measurements of *n* = 5 are plotted. **c** Data for participants with plasma TG ≥ 200 mg/dl (left) and plasma TC ≥ 240 mg/dl (right) were retrieved from the TWB cohort and sequentially stratified by the rs2409674 and rs651821 genotypes. Mean values are indicated. Statistical difference between two groups was determined by the Student’s *t* test. **d** Immunoblot showing APOA5 expression and phosphorylation of GATA4 at serine 105 following treatment of HepG2 cells to phenylephrine. All blots derive from the same experiment and were processed in parallel. **e** Histogram showing the relative rs651821 site enrichment after ChIP with normal mouse IgG or anti-GATA4 using HepG2 and Huh7 cells treated with or without 20 μM phenylephrine for 3 h. **f** Quantification of OA-induced TG level in HepG2 cells co-treated with or without 10 nM silodosin and 20 μM phenylephrine. *P* values and 95% CIs are indicated, and independent measurements of *n* = 6 are plotted.

Given that knockout of *Apoa5* in mice increases plasma TG level, and mutation at *APOA5* gene causes hypertriglyceridemia and elevated TC^19,22^, GATA4 may affect TG and TC homeostasis through APOA5. We treated HepG2 cells with OA, which causes lipid accumulation and steatosis, and monitored changes in the levels of TG and TC. Knockdown of *APOA5* or *GATA4* resulted in significant accumulation of both TG (Fig. 5a) and TC (Fig. 5b) in cells. To confirm the clinical relevance between GATA4-induced *APOA5* upregulation and TG regulation, we divided our TWB cohort into two groups by *GATA4* eQTL. The rs590941-G is carried by relatively few Taiwanese individuals compared with EUR (5.3% in the TWB vs. 29% in EUR). Therefore, for subsequent analysis, we chose another *GATA4* eQTL, namely rs2409674 (Supplementary Fig. 9), with a higher proportion of the minor allele (39.6%) in our population. The results showed that rs651821-TT imparted no significant benefit on plasma TG or TC level for the TWB participants carrying rs2409674-AA/AC, the low-expressing *GATA4* eQTL (Fig. 5c). However, the combination of rs651821-TT and elevated *GATA4* expression (rs2409674-CC) conferred a benefit on plasma TG and TC level (Fig. 5c). These data further confirmed the causal role of rs651821 in the association with hyperlipidemia by mediating allele-specific involvement of GATA4 at *APOA5* expression.

**Fig. 6:**
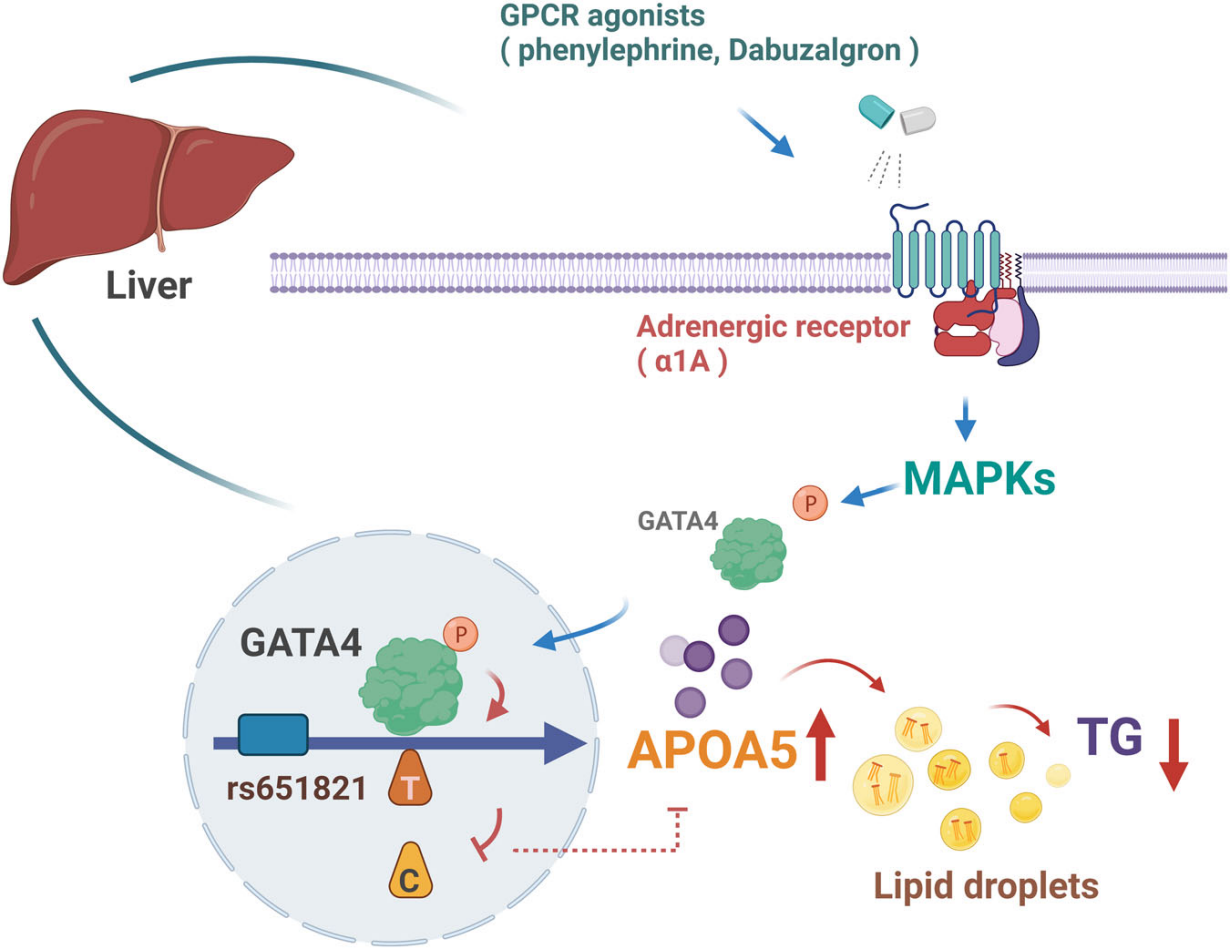
Model of the regulatory mechanism for rs651821 mediating *APOA5* expression through GATA4, thereby promoting TG hydrolysis in liver. The figure was created with BioRender.com.

In cardiac myocytes, GATA4 activity is induced by phenylephrine through phosphorylation at serine 105^23^. Phenylephrine is a nonselective agonist for α_1_-adrenergic receptors^24^. As the α_1A_-adrenergic receptor (encoded by *ADRA1A*) is highly expressed and more abundant than the other α1 subtypes in liver, (Supplementary Fig. 10), we observed phenylephrine promoted APOA5 expression, as well as GATA4 serine 105 phosphorylation, in HepG2 cells (Fig. 5d). This finding result can be explained by ChIP analysis that phenylephrine treatment enhanced GATA4 enrichment at rs651821 in liver cells (Fig. 5e). Moreover, lipid accumulation caused by OA and silodosin, the selective inhibitor of α_1A_-adrenergic receptor, could be alleviated by administration of phenylephrine (Fig. 5f). Collectively, this suggests a therapeutic model for the synergistic effect of APOA5 and GATA4 on TG level, which may help control an individual’s susceptibility to hyperlipidemia and other metabolic abnormalities (Fig. 6).

## Discussion

Given that abnormal levels of TG and TC constitute a threat to human health worldwide, our findings have both etiological importance and translational relevance. Explicitly, we demonstrate a causal role for GATA4 in modulating APOA5 level. Our study suggests standard avenues for demonstrating how to utilize GWAS results to identify cellular pathways that can be modulated via pharmacological means.

Among these hyperlipidemia-associated five loci, a mechanism has been established for only rs780092 (2p23.3), owing to LD with rs780094 (*r*^2^ = 0.45 in EAS of the 1000 Genomes Project); rs780094 facilitates the binding of transcription factor FOXA2 to regulate *GCKR* expression^16^. The rs2954031 (8q24.13) has been explained by its LD-SNP, rs2001844, which was reported as an eQTL for *TRIB1* gene^25^, although how TRIB1 regulates TG remains unclear. The rs2075650 (19q13.32) is located in an intron of *TOMM40*, and rs1501908 (5q33.3) is located between *TIMD4* and *HAVCR1*. However, these two SNPs do not act as the *cis*-eQTL for adjacent genes in GTEx liver tissue. The locus 11q23.3, led by rs662799, is the most significant locus consistently identified. Given that the molecular mechanism by which this locus contributes to metabolic disease remains unclear, our current study as well as several others have attempted to determine the functional significance of rs662799^20,21^, but this SNP has not been definitively linked to the transcription-promoting activity of APOA5. Of special note here is that interferon regulatory factors (IRFs) were predicted to repress *APOA5* through the rs662799-G, but we failed to observe any allele-specific modulation of luciferase activity by IRFs (Supplementary Fig. 1). Apparently, rs662799 is not the causal variant. Instead, another SNP LD with and adjacent to rs662799, namely rs2266788, were then expected. The rs2266788 was reported its interaction with two miRNAs, namely miR-3201 and miR-485-5p^26,27^. These two miRNAs were identified based on their sequence complementary with the rs2266788 region (Supplementary Fig. 11). However, these two miRNAs are actually undetectable in GTEx liver tissue, which argues against their participation in regulating APOA5 level in liver. Additionally, miR-3201 reportedly represses *APOA5* expression through the rs2266788-A, the allele that is thought to be associated with higher APOA5 level^26^. This obviously conflicts with the current paradigm. For miR-485-5p, neither allele of rs2266788 is targeted with high complementarity by the seed region of miR-485-5p (Supplementary Fig. 11), strongly weakening the probability that the miR-485-5p modulates *APOA5* expression. Genetically, rs662799 is in almost complete LD with rs651821 (*r*^2^ = 0.99) but not rs2266788 (*r*^2^ = 0.75). Thus, we propose rs651821 is more likely to be the functional variant within locus 11q23.3.

APOA5 interacts with very low–density lipoprotein (VLDL), the major carrier of TG, and decreases the TG level in circulating VLDL by enhancing lipoprotein lipase activity^28^. As a result, loss-of-function mutations in *APOA5* cause severe hypertriglyceridemia^22,28^. Similarly, mutations in *LPL* (lipoprotein lipase), *APOC2*, *GPIHBP1*, and *LMF1*, and the recently identified transcription factor CREB3L3, are also found to contribute to the development of severe hypertriglyceridemia^6,29^. Although these rare mutations are important, they account for at most 15.2% of the etiology of hypertriglyceridemia, yet >32% of patients having severe hypertriglyceridemia are estimated to be affected by a combination of common variants^6^. Hence, polygenic risk scores, which have been mainly based on EUR, are currently used to predict the development of complex phenotypes, such as hyperlipidemia and abnormal TG and TC levels^6^. However, it should be acknowledged that certain common variants—with rs651821 being a good example—is more prevalent in the EAS (29%) than that in other populations in the world (only 8% in the EUR). That explains why this variant has not been thoroughly investigated in previous GWAS conducted with EUR, and, more critically, why the involvement of GATA4 in regulating *APOA5* as well as plasma TC and TG levels has seldom been appreciated.

Our study showed insufficient GATA4 activity alters the regulation of TG level in cells and in clinical samples. This finding suggests that GATA4 agonist(s), such as phenylephrine, could be developed to treat hyperlipidemia, and exploring the transcriptomic change affected by phenylephrine would be crucial to understand the possible therapeutic action through GATA4 activity. While phenylephrine has shown therapeutic potential to downregulate hepatic TG level in animal models^30^, and, supported by the findings of the present study, phenylephrine may increase blood pressure clinically by promoting vasoconstriction^31^, an unfavorable side effect for hyperlipidemia patients. To overcome this side effect, one α_1A_-adrenergic agonist, Ro 115-1240 (dabuzalgron), has demonstrated no effect on blood pressure and heart rate in clinical study and would expect to be much better choice to treat hyperlipidemia^32,33^. In conclusion, better understanding of the mechanisms underpinning this process is certainly needed to develop personalized preventive and therapeutic strategies for controlling hyperlipidemia and abnormal TC level.

## Methods

### Study cohort

A total of 23,988 samples with genotype and biochemical data were collected from the TWB (Supplementary Table 4). Individuals who had been diagnosed with hyperlipidemia (self-reported) or had a measured TG of ≥200 mg/dl or TC ≥ 240 mg/dl were defined as hyperlipidemia cases, and other individuals who matched the criteria of TG < 150 mg/dl and TC < 200 mg/dl were selected as the control group. Individuals who had intermediate measures were excluded. The discovery stage of the study included 18,419 individuals, among whom 3310 were selected as cases and 6404 as controls. An additional 829 cases and 1833 controls from the remaining non-overlapped 5569 individuals were included in the validation stage. The study was approved by the ethics committee of the institutional review board of Academia Sinica (AS-IRB01-16018) with informed consent that was obtained from each participant in accordance with the institutional requirements and the Declaration of Helsinki principles.

### Genotyping and association analysis

Blood DNA samples from the study participants were genotyped using the Affymetrix TWB SNP chip, as described^18^. For the SNP quality-control process, we selected SNPs with a call rate ≥95%, *P* value for Hardy–Weinberg equilibrium (HWE) of ≥1 × 10^–4^, and minor allele frequency (MAF) ≥ 5%. In total, 597,758 SNPs were selected for the association analysis. Quality-control for genotypes and biochemical measures was performed with Plink software^34^, and odds ratios for individual SNPs associated with hyperlipidemia were calculated using logistic regression with adjustment for the effect of age, sex, body mass index, and principal components. Significance was defined according to the conventional genome-wide significance threshold (5 × 10^–8^), and SNPs located within a ±500 kb region of a significant SNP were considered as a part of that locus. SNPs were further imputed with SHAPEIT and IMPUTE2 software using EAS data from Phase 3 (Version 5) of the 1000 Genomes Project as the reference panel, as described^35^. SNPs with a posterior probability of <0.9, call rate <0.01, MAF < 1%, or HWE *P* value of <1 × 10^–6^ were excluded, and the other SNPs were plotted to visualize the association using LocusZoom^36^.

### Analysis of data from the GTEx portal

The transcripts per million (TPM) normalized RNA sequences data for genes and the expression quantitative trait loci (eQTL) results in liver samples were retrieved from GTEx Analysis V8 (www.gtexportal.org). The statistical significance was determined using the website’s algorithm^37^. The top *cis*-eQTL for *GATA4* was initially selected from 40,414 SNPs that are located within ±1 Mb of the transcription start site of *GATA4*. Because smaller sample sizes restrict minor allele stratification, we excluded those candidate SNPs with MAF <10% (Supplementary Dataset 1). In consequence, rs590941 was the top eQTL with the lowest *P* value of 0.0062. To assess any genetic interaction between rs651821 and rs590941, gene expression data and genotypes for rs651821 and rs590941 in liver-tissue samples from 156 individuals were accessed from GTEx protected data in dbGaP (Database of Genotypes and Phenotypes), and *APOA5* and *GATA4* expression data were transformed to a *z* score before analysis.

### Cell culture

Human liver cell lines HepG2 (RRID:CVCL_0027), cultured in Eagle’s minimum essential medium (#M0643, Sigma–Aldrich), and HA22T (synonyms HA22T/VGH, RRID:CVCL_7046), cultured in DME medium (#D5648, Sigma–Aldrich) supplemented with 0.1 mM non-essential amino acids, were purchased from the Bioresource Collection and Research Center (Hsinchu, Taiwan). Human liver cell lines Hep3B (synonyms Hep3B2.1-7, RRID:CVCL_0326) and Huh7 (RRID:CVCL_0336) were obtained from Dr. Hui-Chun Wang (Kaohsiung Medical University), and Huh6 (RRID:CVCL_4381) was obtained from Dr. Chia-Hung Yen (Kaohsiung Medical University). These cells were cultured in DME medium. Human embryonic kidney 293 T cells (synonyms HEK293T, RRID:CVCL_0063) were as previously described^35^. All cells were maintained in medium supplemented with 10% fetal bovine serum (#26140, Thermo Fisher Scientific). All cell lines were confirmed to be mycoplasma-free and authenticated within the last three years using the short‐tandem repeats profiling method (Promega GenePrint 24 System) by Genelabs (Taipei, Taiwan).

### Treatment and transfection

Oleic acid (OA) sodium salt (#O7501) and phenylephrine (#P6126) were purchased from Sigma–Aldrich, and silodosin (#6663) was obtained from Tocris Bioscience. For the in vitro model of hepatic steatosis, OA was dissolved in 99% methanol as a stock solution^38^. Before treatment, HepG2 cells were washed twice with Hank’s balanced salt solution (#H1387, Sigma–Aldrich) and then incubated in phenol red-free DME medium (#D2902, Sigma–Aldrich) containing 1% bovine serum albumin and 10% charcoal-stripped fetal bovine serum (#12676, Thermo Fisher Scientific).

Cells were transfected with plasmids using TransIT-X2 (Mirus), whereas transfection with small interfering RNAs (siRNAs) utilized Lipofectamine RNAiMAX (Thermo Fisher Scientific). Full-length cDNAs encoding *GATA3*, *GATA4*, *GATA6*, *FOXA1*, *TCF7L2*, *IRF1*, *IRF2* and *IRF3* were amplified from human cell-line cDNAs and cloned into the Myc-tagged pXJ vector, and expression in mammalian cells was assessed via immunoblotting with anti-Myc (1:3000, #M4439, Sigma–Aldrich). The other antibodies used for immunoblotting were: anti-GATA4 (1:500, #MA5-15532), anti-phospho-GATA4 (1:500, #44-948), and anti-APOA5 (1:1500, #MA1-16809) from Thermo Fisher Scientific; anti-actin (1:2000, #A2066), and anti-α-tubulin (1:10000, #T6199) from Sigma–Aldrich. Supplementary Table 5 lists the primer pairs used for plasmid construction. The control siRNA has been described^39^. *GATA4* siRNA pools (#s5603, #s535120 and #s535121) and *APOA5* siRNA (#s528181) were obtained from Thermo Fisher Scientific.

### Luciferase reporter assay

All reporter fragments were amplified using 293T genomic DNA to obtain two distinct genotypes and then cloned into vector pGL4.23 (#E8411, Promega). Supplementary Table 5 lists the primer pairs used for reporter-fragment construction. For reporter analysis, pGL4.23 constructs contained fragments with the major or minor allele of each of rs662799 or rs651821 of the pGL4.23 vector was left empty (control). HepG2 (40,000 cells/well), Huh7 (35,000 cells/well), or Huh6 (15,000 cells/well) in 24-well plates were co-transfected with expression vectors or siRNAs. pRL-tk, which encodes *Renilla* luciferase, was co-transfected as an internal control. After 48 h of incubation, reporter activities were determined using the Dual-Luciferase Reporter Assay System (#E1960, Promega) and normalized to the background activity measured in cells transfected with empty pGL4.23^35,39^. The mean and 95% confidence interval (CI) for each group was calculated based on the pools of four independent experiments, each with four replicates from independent wells. The statistical significance of differences was determined using the Student’s *t* test.

### Mass spectrometry

The 5’-biotinylated oligonucleotide duplexes are indicated in Supplementary Table 5. Briefly, nuclear extract from HepG2 cells was prepared using the NE-PER Nuclear and Cytoplasmic Extraction kit (Thermo Fisher Scientific). The reaction was incubated at room temperature in 500 μl binding buffer containing 10 mM Tris-HCl (pH 7.5), 50 mM KCl, 10 mM MgCl_2_, 10% glycerol, and 1 mM DTT. In each reaction, 500 pmol of a different probe duplex was incubated with 500 μg nuclear extract plus 5 μg poly(dI:dC) (#P4929, Sigma–Aldrich) and 5 μg poly(dA:dT) (#P9764, Sigma–Aldrich). During a 90-min incubation, 30 μl of streptavidin Sepharose beads (#17511301, Cytiva) was added with additional incubation for 30 min. The pulldown matrix coupled to beads was then washed four times with 500 μl wash buffer (20 mM Tris-HCl pH 7.5, 500 mM NaCl, 0.2 mM EDTA). Proteins were eluted and analyzed by liquid chromatography coupled with tandem mass spectrometry (carried out by Biotools Company, New Taipei City, Taiwan). The experiments were performed three times using independently prepared batches of HepG2 nuclear extract, and label-free proteins from the identified peptides were quantified based on normalized peptide-spectra matches (Supplementary Dataset 2).

### Quantitative PCR (qPCR) for gene expression and chromatin immunoprecipitation (ChIP)

Gene expression of *APOA5* or *GATA4* was quantified via reverse-transcription qPCR^35,39^. To determine whether the rs651821 region could serve as an enhancer, mRNA was extracted from HepG2 cells that had been co-transfected using TransIT-X2 with pcDNA3.3-dCas9-VPR or p5w-dCas9-KRAB and a pU6-sgRNA vector encoding sgEGFP, sgRNA1, or sgRNA2 sequence^35^. Supplementary Table 5 lists the primer pairs used for cDNA quantification and sgRNA construction. Relative gene expression was normalized to that for *TBP* using the comparative CT method. Three independent experiments were performed, each with three experimental replicates, and mean and 95% CI values were calculated using the Student’s *t* test.

ChIP was performed using the EZ-Magna ChIP G kit (#17-611, Merck-Millipore) according to previous studies^35,39^. HepG2 (6 × 10^6^ cells/dish) or Huh7 (3 × 10^6^ cells/dish) cells in 100 mm dishes were fixed in 1% formaldehyde, and DNA was sonicated with Bioruptor Pico (Diagenode). Sheared DNA was then immunoprecipitated with 1 μg ChIP-grade antibody: anti-GATA3 (#ab199428, Abcam) or anti-GATA4 (#MA5-15532). Normal rabbit or mouse IgG served as the control antibody. Immunoprecipitated DNA was eluted and then quantified via SYBR qPCR (#A25741, Thermo Fisher Scientific). For those cells overexpressing Myc-tagged GATA family members, sheared DNA was immunoprecipitated with anti-Myc (#M4439). Allele-specific quantification of eluted DNA in 293 T cells was determined with TaqMan™ Fast Advanced Master Mix (#4444557, Thermo Fisher Scientific) with the specific fluorescence probe set described in Supplementary Table 5.

### Measurement of intracellular TG and TC

TG and TC content in steatotic HepG2 cells was determined with the Triglyceride-Glo Assay (#J3160, Promega) and Cholesterol/Cholesterol Ester-Glo Assay, (#J3190, Promega), respectively. Briefly, HepG2 cells that had been subjected to siRNA-mediated mRNA knockdown were seeded in a 96-well plate (3000 cells/well) and treated with 0.5 mM OA for 24 h. The medium was removed, and cells were washed twice by Hank’s balanced salt solution and lysed with 30 μl buffer included in the kits. The concentration of each of TG and TC was calculated based on standard curves. Mean and 95% CI values for each experimental group were calculated from five independent wells, and three independent experiments were performed. The statistical significance was determined using the Student’s *t* test.

### Reporting summary

Further information on research design is available in the Nature Research Reporting Summary linked to this article.

## Supplementary information

Supplementary Information

Supplementary Data 1

Reporting Summary

## Data Availability

The expression vectors generated during the current study are available from the corresponding author on request. The GWAS summary statistics are deposited in the GWAS catalogue https://www.ebi.ac.uk/gwas/ (accession numbers: GCST90090994) and are openly available in figshare at 10.6084/m9.figshare.14897097. Individual data are used under license for the current study, and so are not publicly available. Data are, however, available directly from the Taiwan Biobank (biobank@gate.sinica.edu.tw) pending permission from the Ministry of Health and Welfare, Taiwan. Mass spectrometry was carried out by Biotools Company and the proteomics RAW files are unavailable. However the quantification of label-free proteins based on normalized peptide-spectra matches are available within the article and its Supplementary Dataset 2. Public datasets that support the findings of this study are available from GTEx portal, https://www.gtexportal.org/home/datasets; Genomics of Drug Sensitivity in Cancer Project, https://www.cancerrxgene.org/downloads/bulk_download; Cancer Cell Line Encyclopedia, https://portals.broadinstitute.org/ccle/data; and in ArrayExpress, https://www.ebi.ac.uk/arrayexpress/experiments/E-MTAB-2706/.
