# Supplementary material for: A common variant in 11q23.3 associated with hyperlipidemia is mediated by the binding and regulation of GATA4
Source: NPJ Genom Med. 2022 Jan 19;7:4. doi: 10.1038/s41525-021-00279-5 (PMC8770627; doi:10.1038/s41525-021-00279-5)

A common variant in 11q23.3 associated with hyperlipidemia is mediated by the binding and regulation of GATA4

Authors: Wen-Cheng Chou[1], Wei-Ting Chen[1], Chen-Yang Shen[1,2,]*

Affiliations

[1]Institute of Biomedical Sciences, Academia Sinica, Taipei, Taiwan

[2]College of Public Health, China Medical University, Taichung, Taiwan

Supplementary information

Supplementary Tables 1-3, 5-6……….……….………...………...……………….…..P2

Supplementary Figures 1-11…...……………………………..……………...…...P8

Supplementary Tables

Supplementary Table 1. Comparison of MAFs of five lead variants in different ethnic populations

Supplementary Table 2. Comparison of association results for lipid loci reported in previous East Asian analysis.

Supplementary Table 3. *Cis*-eQTL analysis for susceptibility SNPs and their adjacent apolipoprotein genes

Supplementary Table 4. List of variants and *cis*-eQTL analyses within ±1 Mb of the transcription start site of GATA4. (separated file)

Supplementary Table 5. Characteristics of the study population from Taiwan Biobank.

Supplementary Table 6. Primer sequences used in the study.

Supplementary Table 7. Transcription factors identification and quantification in biotin pulldown experiments. (separated file)

Supplementary Table 1. Comparison of MAFs of five lead variants in different ethnic populations

SNP	Chr	Position	Ref	Alt	TWB		1000 Genomes Project				
					Array*	Seq**	EAS	AFR	AMR	EUR	SAS
rs780092	2	27743154	A	G	0.36	0.35	0.34	0.24	0.14	0.17	0.25
rs1501908	5	156398169	C	G	0.27	0.26	0.27	0.68	0.30	0.35	0.29
rs2954031	8	126491733	T	G	0.47	0.47	0.46	0.62	0.60	0.57	0.68
rs662799	11	116663707	A	G	0.28	0.27	0.29	0.12	0.15	0.083	0.19
rs2075650	19	45395619	A	G	0.082	0.070	0.097	0.13	0.11	0.13	0.12

Abbreviations: MAFs, minor allele frequencies; SNP, single-nucleotide polymorphism; Chr, chromosome; Ref, reference allele; Alt, alternative allele; TWB, Taiwan Biobank database; Seq, sequence; EAS, East Asian; AFR, African; AMR, American; EUR, European; SAS, South Asian.

*, MAFs obtained from current study cohort.
**, MAFs obtained by sequence-based method.

Supplementary Table 2. Comparison of association results for lipid loci reported in previous East Asian analysis.

SNP	Chr	Position	Nearest gene	Ref	Alt	East Asian Meta-Analysis*						Taiwan Biobank Analysis					Replicate (Y or N)
						Trait	N	MAF	Beta	SE	*P*	N	MAF	Beta	SE	*P*	
rs599839	1	109822166	SORT1	A	G	TC	32013	0.07	-0.168	0.016	4.03×10^{-25}	9711	0.08	-0.204	0.061	7.75×10^{-4}	Y
rs780094	2	27741237	GCKR	C	T	TG	27651	0.52	0.105	0.009	4.35×10^{-33}	9708	0.48	0.196	0.033	1.65×10^{-9}	Y
rs780092	2	27743154	GCKR	A	G	TC	32010	0.33	-0.053	0.009	1.14×10^{-9}	9694	0.36	-0.256	0.035	1.09×10^{-13}	Y
rs12916	5	74656539	HMGCR	C	T	TC	32016	0.47	-0.079	0.008	2×10^{-22}	9706	0.46	-0.119	0.033	2.62×10^{-4}	Y
rs7775698	6	135418635	HBS1L	C	T	TC	20198	0.29	-0.076	0.011	2.78×10^{-11}	9689	0.23	-0.02	0.039	0.603	N
rs13233571	7	72971231	MLXIPL	C	T	TG	27652	0.11	-0.119	0.014	2.44×10^{-17}	9703	0.1	-0.191	0.056	7.33×10^{-4}	Y
rs2001945	8	126477978	TRIB1	C	G	TG	27645	0.42	0.065	0.009	1.16×10^{-13}	9708	0.44	0.224	0.033	5.66×10^{-12}	Y
rs17482753	8	19832646	LPL	G	T	TG	25888	0.11	-0.154	0.014	8.97×10^{-26}	9701	0.1	-0.276	0.057	1.2×10^{-6}	Y
rs10503669	8	19847690	LPL	C	A	TG	25795	0.11	-0.154	0.014	1.16×10^{-25}	9680	0.1	-0.279	0.057	9.33×10^{-7}	Y
rs1883025	9	107664301	ABCA1	C	T	TC	31777	0.25	-0.073	0.009	1.44×10^{-14}	9707	0.23	-0.161	0.038	2.87×10^{-5}	Y
rs579459	9	136154168	ABO	T	C	TC	31962	0.35	0.059	0.009	3.77×10^{-10}	9704	0.18	0.175	0.042	2.7×10^{-5}	Y
rs964184	11	116648917	ZNF259	C	G	TG	27594	0.22	0.216	0.01	1.81×10^{-96}	9700	0.21	0.454	0.04	7.15×10^{-30}	Y
rs662799	11	116663707	APOA5	A	G	TG	18857	0.27	0.282	0.011	1.1×10^{-127}	9698	0.28	0.59	0.037	5.9×10^{-58}	Y
rs1077834	15	58723479	LIPC	T	C	TG	17335	0.41	0.084	0.011	2.45×10^{-13}	9698	0.38	0.108	0.033	1.19×10^{-3}	Y
rs1800774	16	57015545	CETP	C	T	TC	31808	0.12	-0.069	0.012	3.67×10^{-8}	9705	0.14	-0.03	0.047	0.516	N
rs9958734	18	47118398	LIPG	T	C	TC	20453	0.39	0.092	0.011	2.6×10^{-16}	9694	0.41	0.088	0.033	7.57×10^{-3}	Y

Association results for lead loci identified from the discovery stage of the East Asian meta-analysis. Only results for the loci available in the study are present and compared. All previously reported SNPs showed the same direction of effect for the alternative allele between two studies, and 14 of 16 SNPs were successfully replicated with $P < 0.05$ in the study.

Abbreviations: SNP, single-nucleotide polymorphism; Chr, chromosome; Ref, reference allele; Alt, alternative allele; N, number; MAF, minor allele frequency; SE, standard error; TC, total cholesterol; TG, triglyceride.

*, obtained from ref. 9 of the main text.

Supplementary Table 3. *Cis*-eQTL analysis for susceptibility SNPs and their adjacent apolipoprotein genes.

SNP	Allele	Gene	Effect Size	P_{eQTL}
rs662799	A/G	*APOA1*	-0.16	0.16
(*n* = 207)		*APOC3*	-0.058	0.48
		APOA4	-0.14	0.30
		APOA5	–0.25	0.011
rs651821	T/C	*APOA1*	-0.20	0.06
(*n* = 207)		*APOC3*	-0.12	0.13
		APOA4	–0.23	0.067
		APOA5	–0.24	0.012

Abbreviations: SNP, single-nucleotide polymorphism; eQTL, expression quantitative trait locus

Supplementary Table 5. Characteristics of the study population from Taiwan Biobank.

	Cohort (N = 23988)
Age (year)	49.28 ± 10.97
Sex (% female)	50.5%
BMI (kg/m^2)	24.28 ± 3.585
Triglyceride (mg/dL)	112.5 ± 81.68
Cholesterol (mg/dL)	193.5 ± 34.22
LDL (mg/dL)	121.6 ± 30.94
HDL-C (mg/dL)	53.53 ± 13.03
HbA1c (%)	5.646 ± 0.6483
WHR	0.8701 ± 0.06709

Abbreviations: BMI, body mass index; HDL, high-density lipoprotein cholesterol; LDL, low-density lipoprotein cholesterol; WHR, waist-hip ratio.

Supplementary Table 6. Primer sequences used in the study.

Target	Nucleotide Sequence 5'→3'	Forward/ Reverse
Cloning		
GATA3 (NM_002051.2)	CAGGGATCCGCCATGGAGGTGACGGCGG	F
	TAGCTCGAGCTAACCCATGGCGGTGACC	R
GATA4 (NM_001308093.1)	GCAAGATCTATGTATCAGAGCTTGGCCATGG	F
	AGACTCGAGATTACGCAGTGATTATGTCCCC	R
GATA6 (NM_005257.5)	GCCGGATCCTGGATGGCCTTGACTGACGGC	F
	TGTCTCGAGCGCTGCACAAAAGCAGACACGAG	R
FOXA1(NM_004496.3)	GGTGGATCCAGGATGTTAGGAACTG	F
	TCCCTCGAGCTAGGAAGTGTTTAGGAC	R
TCF7L2 (NM_001146274.1)	GAAGGATCCATGCCGCAGCTGAACGGCGGTGGAGGG	F
	ACGCTCGAGCTATTCTAAAGACTTGGTGACGAG	R
IRF1 (NM_002198.2)	AGAGGATCCATGCCCATCACTCGGATGCGCATG	F
	GGGCTCGAGGGGCCCTGCTACGGTGCACAG	R
IRF2 (NM_002199.3)	GAGGGATCCATGCCGGTGGAAAGGATGCGCATG	F
	AGAGTCGACGGCTTAACAGCTCTTGACGCGGGC	R
IRF3 (NM_001571.5)	GTAAAGCTTACCATGGGAACCCCAAAGCCAC	F
	AGGCTCGAGGGCTCAGCTCTCCCCAGGG	R
Reporter		
Fragment A	TGCGGTACCTGTAGTGAAGCTTTCAGGG	F
	ACAGGATCCTCTGGCCAGCCTCCACCC	R
Fragment B	CCTGGTACCAGGGCACTCATTAACCC	F
	ACAGGATCCTCTGGCCAGCCTCCACCC	R
Fragment C	ATGGGTACCGTAGACGGAGTGGGTGTGTCATC	F
	ATACTCGAGGGCCCTGAGCCTCTGGCACC	R
Fragment D	ATTGGTACCAGATGCATTTAGGACCAAGA	F
	CTTCTCGAGACAATCCTGGAACAAGCAAG	R
QPCR		
APOA5	CTGGCTCTTCTTTCAGCGTTT	F
	CAGAGGCCTCAGCTTTTCCA	R
GATA4	TGGGACGGGTCACTATCTGT	F
	TTTGGATCCCCTCTTTCCGC	R
sgRNA		
sgEGFP	CACCGCGAGGTATTCGGCTCCGCG	F
	AAACCGCGGAGCCGAATACCTCGC	R
sgRNA-1	CACCGCATGGCTGCCGTGCTCACC	F
	AAACGGTGAGCACGGCAGCCATGC	R
sgRNA-2	CACCGTGCTCTGAGAAGACAGGTGG	F
	AAACCCACCTGTCTTCTCAGAGCAC	R
ChIP		
rs651821 (SYBR)	CACGTTGAAGTCAGGGTCGG	F
	AGGTCAGTCCTCTTGAGCCC	R
rs651821 (Taqman)	GCACGGCAGCCATGCT	F
	TCGTCTCCTTCTTCCCCTAACC	R
	CCATTATCTGCTCTGAG (5' FAM; 3' MGB)	
	CCATTACCTGCTCTGA (5' HEX; 3' MGB)	
Pulldown		
rs651821_C allele	AGCACGGCAGCCATGCTTGCCATTA**C**CTGCTCTGAGAAGACAGGTGGAGGG	F
	CCCTCCACCTGTCTTCTCAGAGCAG**G**TAATGGCAAGCATGGCTGCCGTGCT	R
rs651821_T allele	AGCACGGCAGCCATGCTTGCCATTA**T**CTGCTCTGAGAAGACAGGTGGAGGG	F
	CCCTCCACCTGTCTTCTCAGAGCAG**A**TAATGGCAAGCATGGCTGCCGTGCT	R
rs651821_deletion	AGCACGGCAGCCATGCTTGCCACTCTGAGAAGACAGGTGGAGGG	F
	CCCTCC ACCTGTCTTCTCAGAGTGGCAAGCATGGCTGCCGTGCT	R

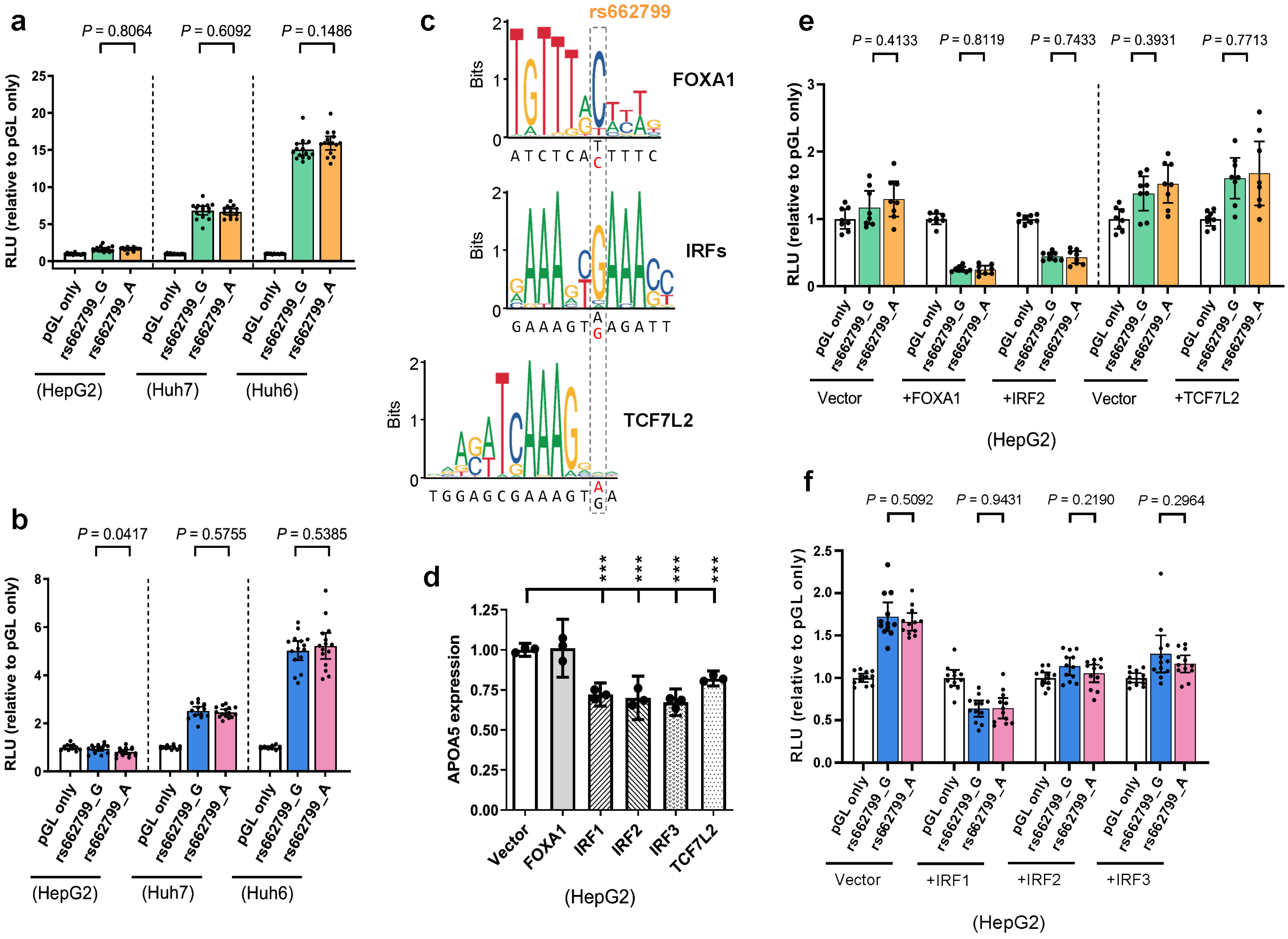

a
P = 0.8064
P = 0.6092
P = 0.1486
RLU (relative to pGL only)
pGL only
rs662799_G
rs662799_A
(HepG2)
(Huh7)
(Huh6)
b
P = 0.0417
P = 0.5755
P = 0.5385
RLU (relative to pGL only)
(HepG2)
(Huh7)
(Huh6)
c
rs662799
Bits
FOXA1
ATCTCA T/C TTTC
IRFs
GAAAGT A/G AGATT
TCF7L2
TGGAGCGAAAGT A/G A
d
APOA5 expression
Vector
FOXA1
IRF1
IRF2
IRF3
TCF7L2
***
(HepG2)
e
P = 0.4133
P = 0.8119
P = 0.7433
P = 0.3931
P = 0.7713
RLU (relative to pGL only)
Vector
+FOXA1
+IRF2
Vector
+TCF7L2
(HepG2)
f
P = 0.5092
P = 0.9431
P = 0.2190
P = 0.2964
RLU (relative to pGL only)
Vector
+IRF1
+IRF2
+IRF3
(HepG2)

Supplementary Fig 1. Functional analysis of rs662799 with respect to *APOA5* regulation. **a** Reporter analysis for plasmid pGL or plasmids containing element C (see Figure 2C) with different rs662799 genotypes in HepG2 cells. RLU, relative luminescence units. **b** Reporter analysis for pGL or plasmids containing element D (see Figure 2C) with different rs662799 genotypes in HepG2 cells. *P* values and 95% CIs for the results in **a** and **b** are indicated; pools of three independent luciferase experiments of n = 4 are shown as dot plots. **c** Predicted consensus motif of transcription factors that were matched to rs662799. Sequence logos were retrieved from the JASPAR 2020 database (http://jaspar.genereg.net/). **d** Histogram showing relative *APOA5* expression in HepG2 cells overexpressing different transcription factors, as determined by qPCR. $^{***}P <$ 0.001. **e** Reporter analysis of element C with different rs662799 genotypes in HepG2 cells transfected with empty vector or a vector expressing one of the indicated transcription factors. **f** Reporter analysis of element D with different rs662799 genotypes in HepG2 cells transfected with empty vector or a vector expressing one of the indicated IRFs. *P* values and 95% CIs for the results in **e** and **f** are indicated; pools of two independent luciferase experiments of n = 4 are shown as dot plots.

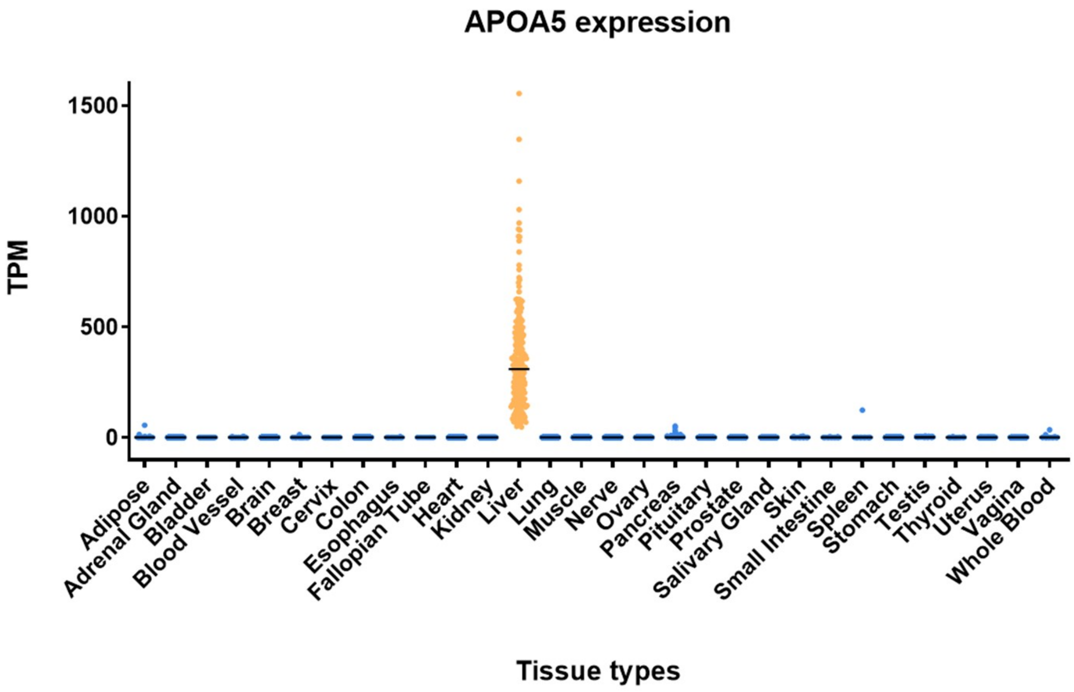

Supplementary Fig 2. Expression profile of *APOA5*. Distribution of *APOA5* in different GTEx tissues sorted alphabetically. Median values are indicated.

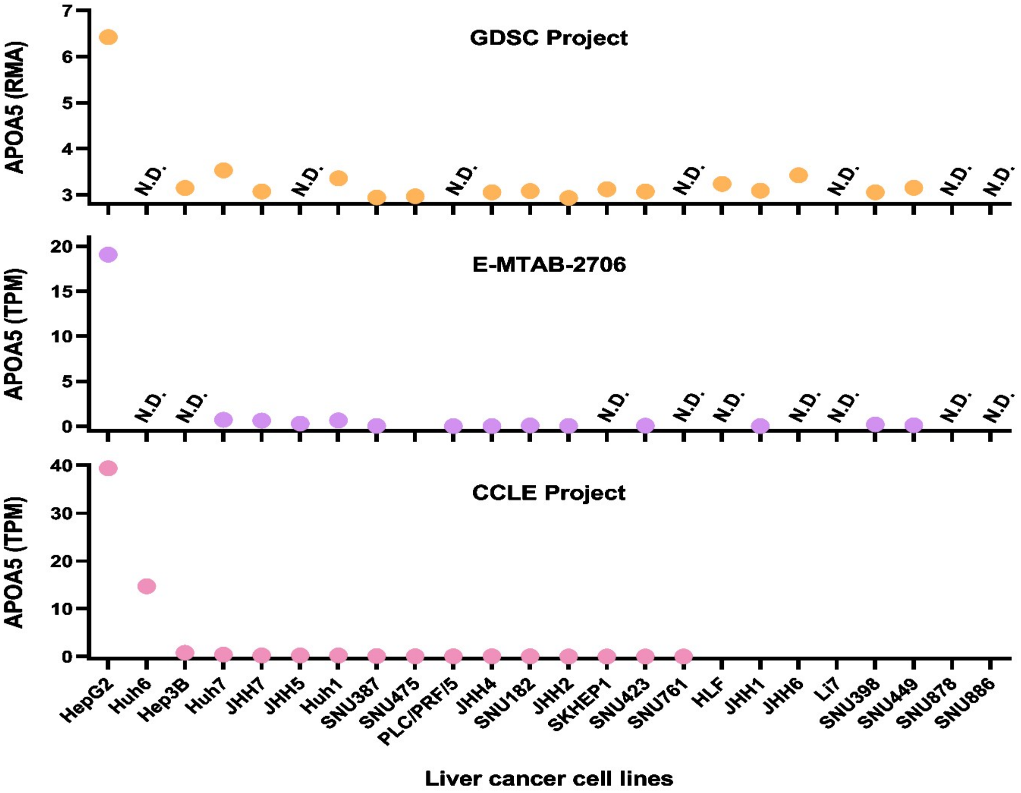

Supplementary Fig 3. Evaluation of *APOA5* expression in different liver cell lines. Upper: Affymetrix Human Genome U219 Array-based expression data (RMA-normalized) obtained from the Genomics of Drug Sensitivity in Cancer Project (https://www.cancerrxgene.org/). Middle: Sequencing-based expression data obtained from the E-MTAB-2706 dataset in ArrayExpress. *APOA5* expression in each cell line is shown after normalization with the TPM. Lower: TPM-normalized expression data obtained from the 2019 Cancer Cell Line Encyclopedia project of The Broad Institute (https://portals.broadinstitute.org/ccle). N.D., not determined in the dataset.

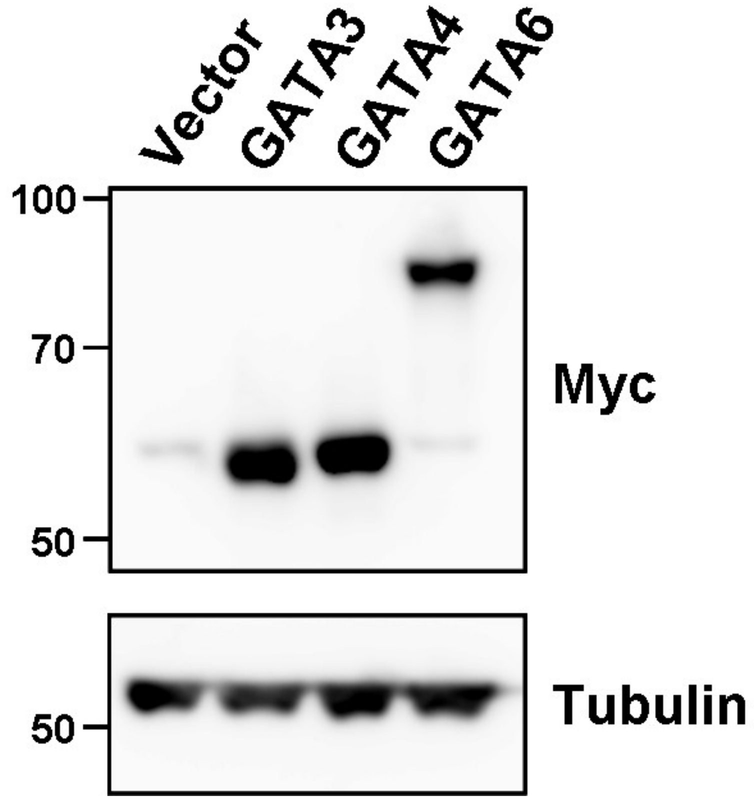

Supplementary Fig 4. Expression test for GATA proteins in 293T cells. 293T cells were transfected for 48 h with Myc-tagged empty pXJ vector or pXJ encoding GATA3, GATA4, or GATA6. Cells were harvested, and GATA expression was detected by immunoblotting.

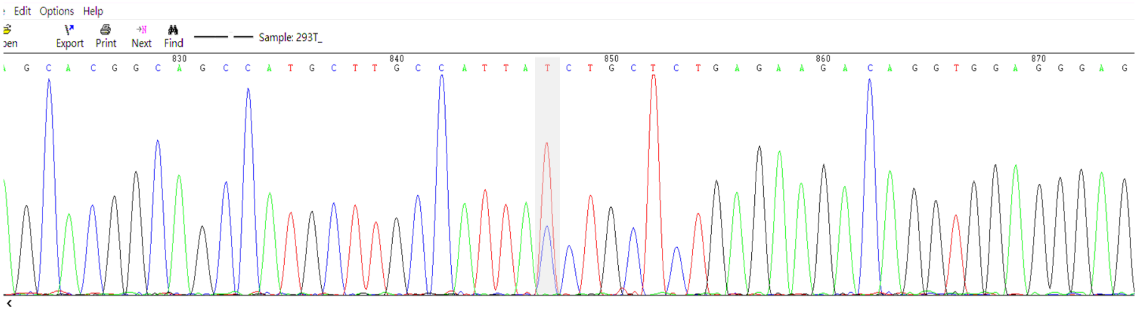

Supplementary Fig 5. Sanger sequencing analysis of the rs651821 genotype in 293T cells.

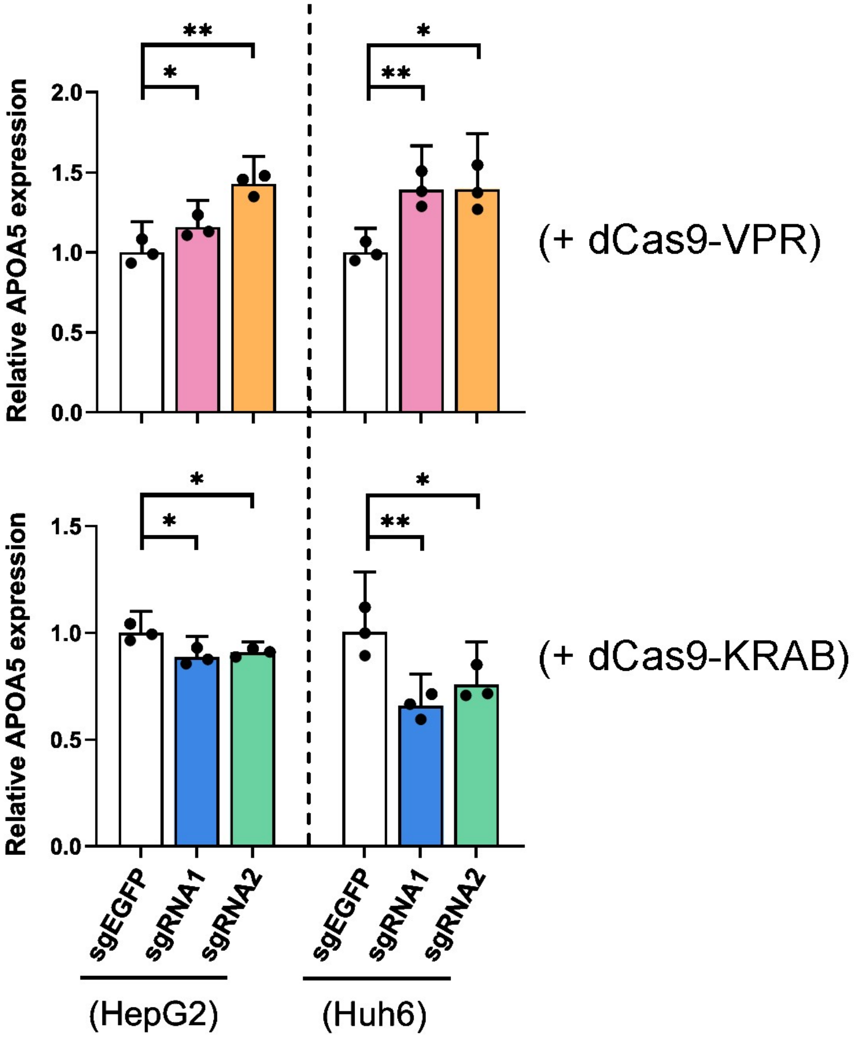

Supplementary Fig 6. Regulation of *APOA5* expression by CRISPRa and CRISPRi technology. Quantitative PCR assays were conducted to compare relative *APOA5* expression in HepG2 (left) and Huh6 (right) cells transfected with control sgRNA (sgEGFP) or one of two distinct sgRNAs targeting the rs651821-adjacent region (sgRNA1 and sgRNA2). Cells were co-transfected with vector dCas9-VPR or dCas9-KRAB for the CRISPRa analysis and CRISPRi analysis, respectively. *P* values between two groups (Student's t-test) and 95% CIs are indicated; $*P < 0.05$; $**P < 0.01$.

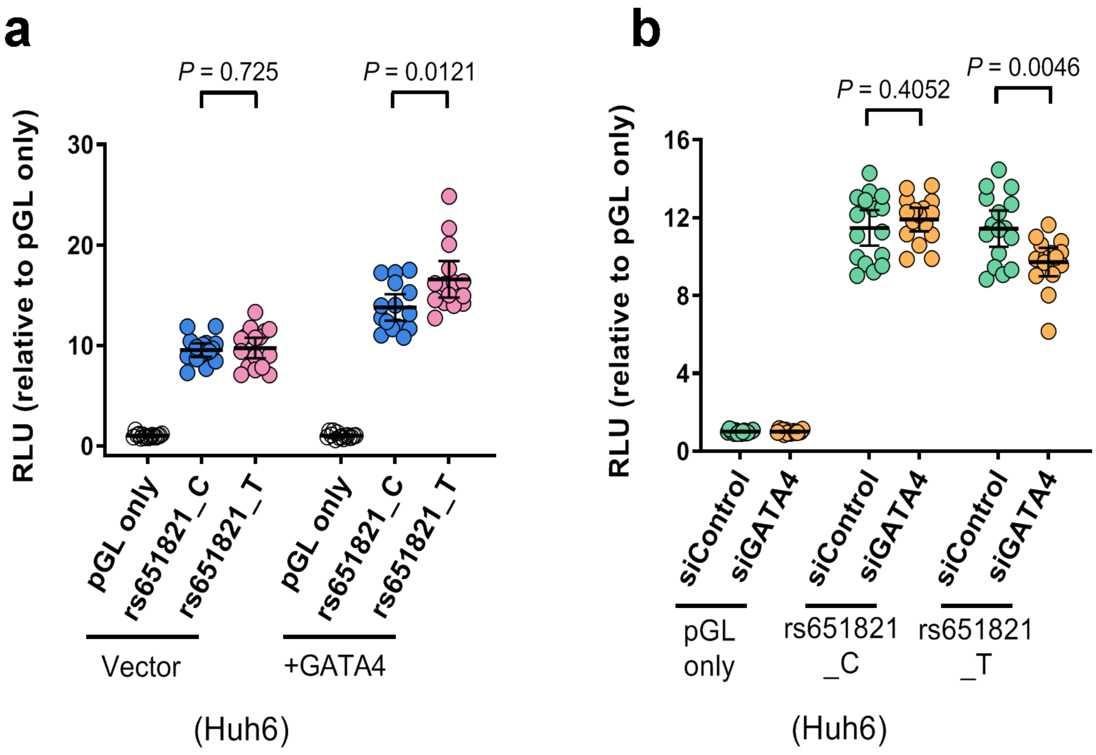

Supplementary Fig 7. Allele-specific reporter activity of the rs651821 site requires GATA4. Reporter analysis of rs651821-containing fragments in Huh6 cells transfected with **a** empty vector or vector overexpressing GATA4, or **b** a control siRNA or *GATA4* knockdown. RLU, relative luminescence units. *P* values and 95% CIs for the results are indicated; pools of four independent luciferase experiments of n = 4 are shown as dot plots.

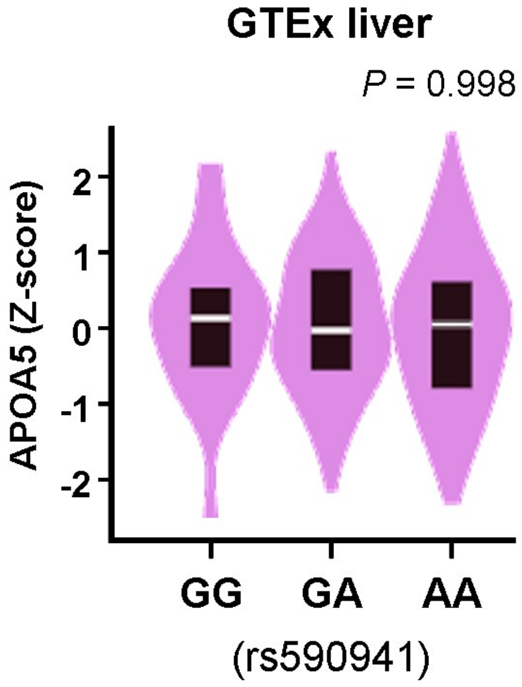

Supplementary Fig 8. *Trans*-eQTL analysis of *APOA5* and rs590941, the *GATA4* *cis*-eQTL in GTEx liver tissue.

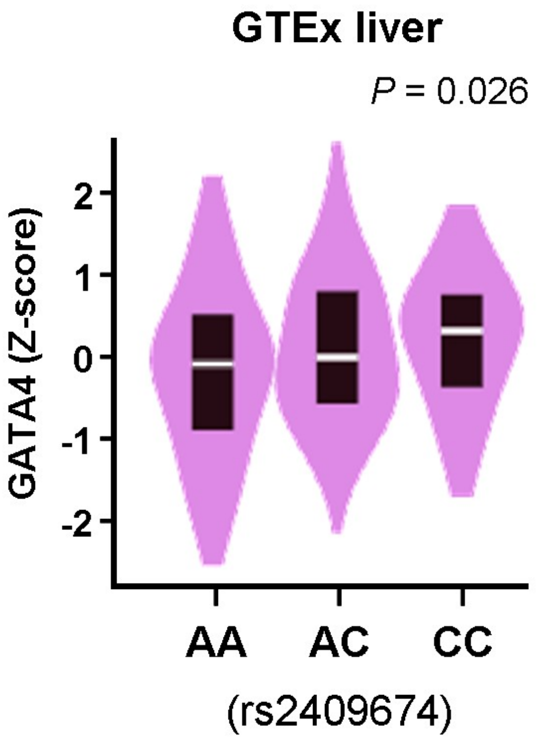

Supplementary Fig 9. Rs2409674 acts as a *GATA4 cis*-eQTL in GTEx liver tissue.

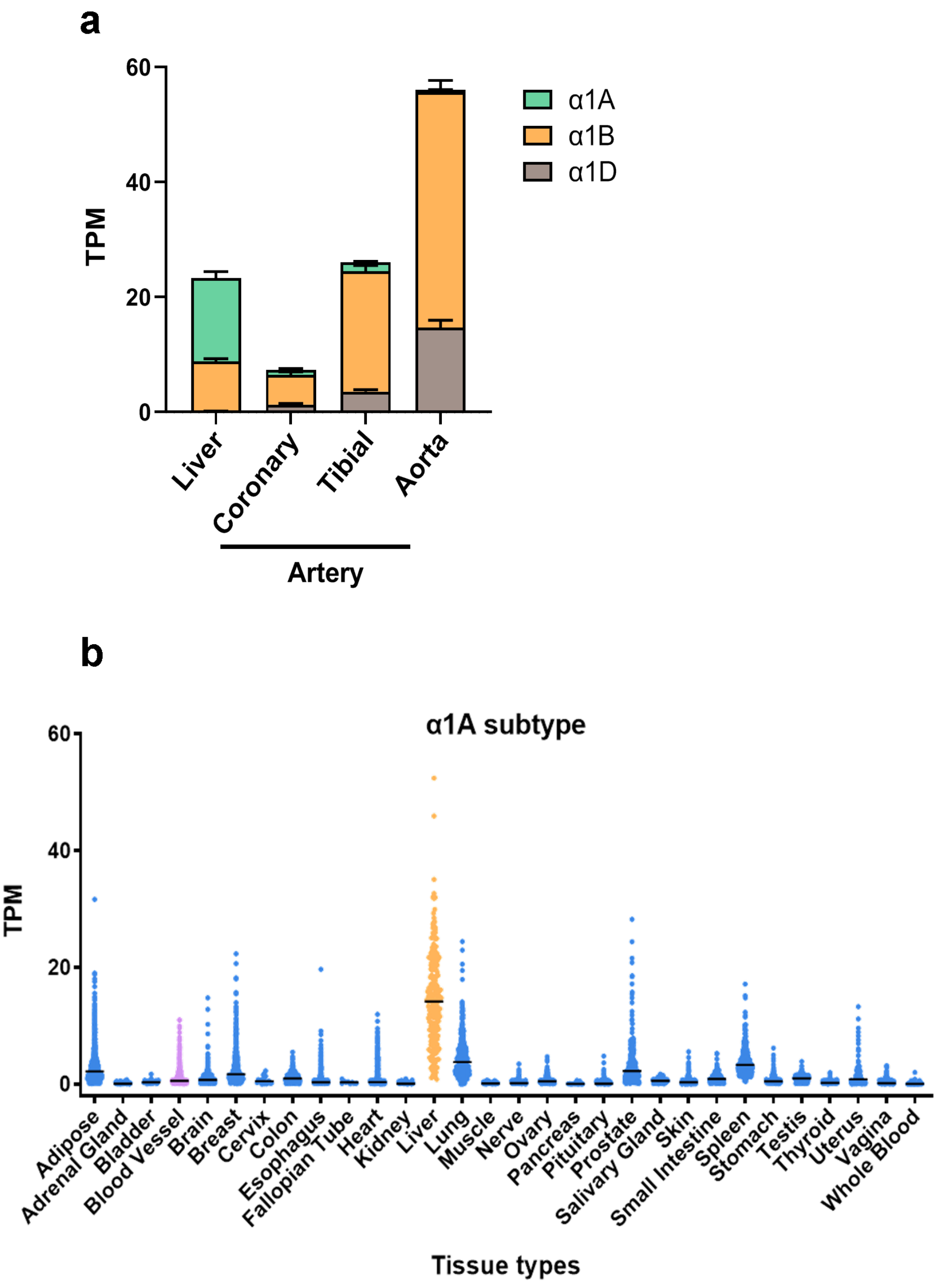

Supplementary Fig 10. Abundance of α_1-adrenergic receptors mRNA in liver. **a** Relative mRNA of each of α_1-adrenergic receptors in GTEx liver tissue and three kinds of blood vessels of GTEx. The percentages of α_{1A} subtype among α_1-adrenergic receptors were indicated (upper). **b** Differential expression of α_{1A} subtype in all GTEx tissues sorted alphabetically. Median values are indicated.

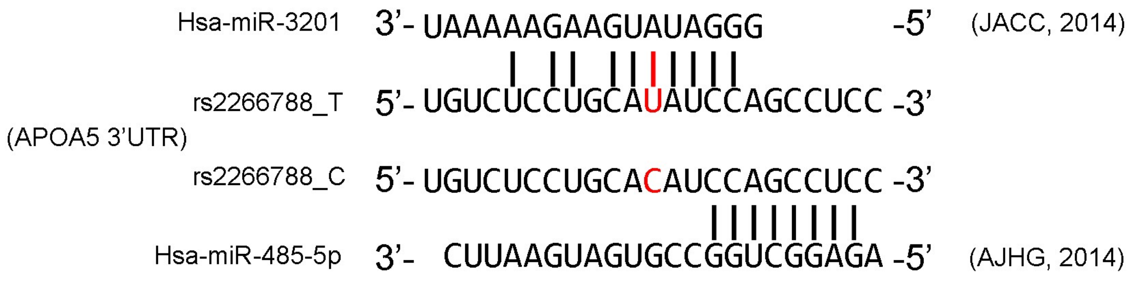

Supplementary Fig 11. Potential base pairing between rs2266788 and two different miRNAs. Details are described at the Discussion. The SNP denoted as rs2266788 is colored in red, and the vertical lines indicate Watson-Crick base pairs between rs2266788 and the seed sequence of the two miRNAs.

Figure 5d: uncropped original blots

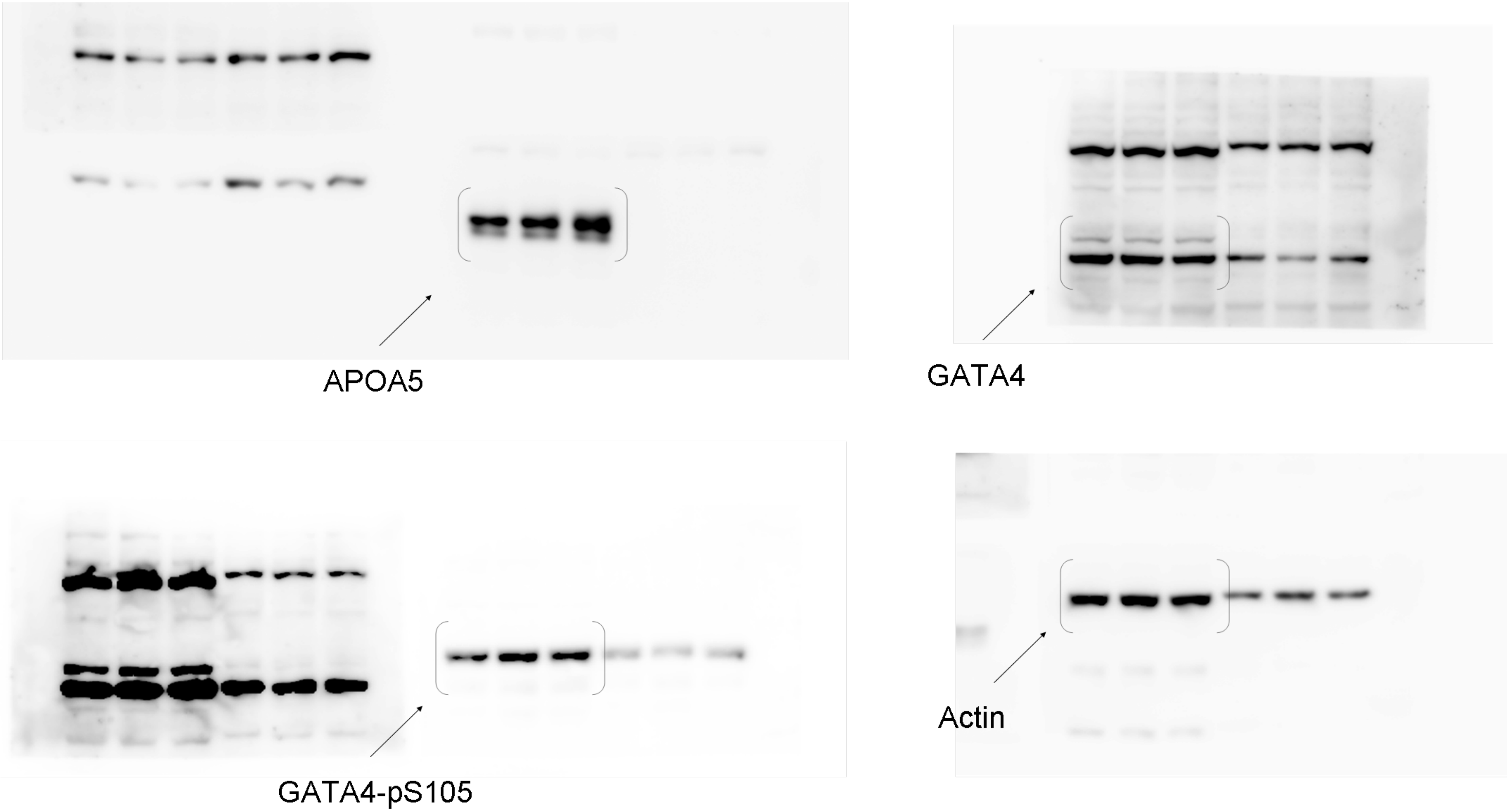

Supplement: Supplementary file 1 — Supplementary Information [file 41525_2021_279_MOESM1_ESM.pdf]
