# Supplementary material for: A common variant in 11q23.3 associated with hyperlipidemia is mediated by the binding and regulation of GATA4
Source: NPJ Genom Med. 2022 Jan 19;7:4. doi: 10.1038/s41525-021-00279-5 (PMC8770627; doi:10.1038/s41525-021-00279-5)
Supplement: Supplementary file 3 — Reporting Summary [file 41525_2021_279_MOESM3_ESM.pdf]

nature portfolio

Corresponding author(s): Chen-Yang Shen

Last updated by author(s): Jul 2, 2021

Reporting Summary

Nature Portfolio wishes to improve the reproducibility of the work that we publish. This form provides structure for consistency and transparency in reporting. For further information on Nature Portfolio policies, see our Editorial Policies and the Editorial Policy Checklist.

Statistics

For all statistical analyses, confirm that the following items are present in the figure legend, table legend, main text, or Methods section.

n/a	Confirmed	
☐	☒	The exact sample size (n) for each experimental group/condition, given as a discrete number and unit of measurement
☐	☒	A statement on whether measurements were taken from distinct samples or whether the same sample was measured repeatedly
☐	☒	The statistical test(s) used AND whether they are one- or two-sided<br>*Only common tests should be described solely by name; describe more complex techniques in the Methods section.*
☐	☒	A description of all covariates tested
☒	☐	A description of any assumptions or corrections, such as tests of normality and adjustment for multiple comparisons
☐	☒	A full description of the statistical parameters including central tendency (e.g. means) or other basic estimates (e.g. regression coefficient) AND variation (e.g. standard deviation) or associated estimates of uncertainty (e.g. confidence intervals)
☐	☒	For null hypothesis testing, the test statistic (e.g. F, t, r) with confidence intervals, effect sizes, degrees of freedom and P value noted<br>*Give P values as exact values whenever suitable.*
☒	☐	For Bayesian analysis, information on the choice of priors and Markov chain Monte Carlo settings
☒	☐	For hierarchical and complex designs, identification of the appropriate level for tests and full reporting of outcomes
☒	☐	Estimates of effect sizes (e.g. Cohen's d, Pearson's r), indicating how they were calculated

Our web collection on statistics for biologists contains articles on many of the points above.

Software and code

Policy information about availability of computer code

Data collection	Datasets in this study were obtained from Taiwan Bank and publicly available websites.
Data analysis	QC for genotypes and biochemical measures was performed with Plink. Association analysis was conducted using R.

For manuscripts utilizing custom algorithms or software that are central to the research but not yet described in published literature, software must be made available to editors and reviewers. We strongly encourage code deposition in a community repository (e.g. GitHub). See the Nature Portfolio guidelines for submitting code & software for further information.

Data

Policy information about availability of data

All manuscripts must include a data availability statement. This statement should provide the following information, where applicable:

- Accession codes, unique identifiers, or web links for publicly available datasets
- A description of any restrictions on data availability
- For clinical datasets or third party data, please ensure that the statement adheres to our policy

Individual data from Taiwan Biobank are available directly from the Taiwan Biobank (biobank@gate.sinica.edu.tw) pending permission from the Ministry of Health and Welfare, Taiwan. Individual data from GTEx protected data are available from dbGaP (https://www.gtexportal.org/home/protectedDataAccess). Mass spectrometry and eQTL data that support the findings of this study are available within the paper and its supplementary files.

March 2021

Field-specific reporting

Please select the one below that is the best fit for your research. If you are not sure, read the appropriate sections before making your selection.

☒ Life sciences ☐ Behavioural & social sciences ☐ Ecological, evolutionary & environmental sciences

For a reference copy of the document with all sections, see nature.com/documents/nr-reporting-summary-flat.pdf

Life sciences study design

All studies must disclose on these points even when the disclosure is negative.

Sample size	GWAS: 18419 individuals.<br>GTEx: sample size restricted to the data available at databases.<br>Reporter assay: 16<br>TG/TC assay: 5<br>ChIP-PCR: 4<br>RT-qPCR: 3
Data exclusions	No data was excluded.
Replication	GWAS result was replicated using non-overlapped 5569 individuals. Cell-based experiments were performed in three independents
Randomization	Not relevant to this study.
Blinding	Not relevant to this study.

Reporting for specific materials, systems and methods

We require information from authors about some types of materials, experimental systems and methods used in many studies. Here, indicate whether each material, system or method listed is relevant to your study. If you are not sure if a list item applies to your research, read the appropriate section before selecting a response.

Materials & experimental systems

n/a	Involved in the study	
☐	☒	Antibodies
☐	☒	Eukaryotic cell lines
☒	☐	Palaeontology and archaeology
☒	☐	Animals and other organisms
☐	☒	Human research participants
☒	☐	Clinical data
☒	☐	Dual use research of concern

Methods

n/a	Involved in the study	
☒	☐	ChIP-seq
☒	☐	Flow cytometry
☒	☐	MRI-based neuroimaging

Antibodies

Antibodies used	anti-GATA4 (#MA5-15532), anti-phospho-GATA4 (#44-948), and anti-APOA5 (#MA1-16809) from Thermo Fisher Scientific; anti-actin (#A2066), anti-α-tubulin (#T6199), and anti-Myc (#M4439) from Sigma-Aldrich.
Validation	Statements of antibodies are on manufacturer's website. anti-Myc used in ChIP and immunoblot was validated by Myc-Tag expression vectors in Figure S4.

Eukaryotic cell lines

Policy information about cell lines

Cell line source(s)	HepG2 (RRID:CVCL_0027) and HA22T (RRID:CVCL_7046) were purchased from the Bioresource Collection and Research Center (Hsinchu, Taiwan). Hep3B (RRID:CVCL_0326) and Huh7 (RRID:CVCL_0336) were obtained from Dr. Hui-Chun Wang (Kaohsiung Medical University). Huh6 (RRID:CVCL_4381) was obtained from Dr. Chia-Hung Yen (Kaohsiung Medical University). 293T (RRID:CVCL_0063) was from Dr. Sheau-Yann Shieh (Academia Sinica)
Authentication	All cell lines were confirmed to be mycoplasma-free and authenticated within the last three years using the short-tandem repeats profiling method (Promega GenePrint 24 System) by Genelabs (Taipei, Taiwan)
Mycoplasma contamination	All cells were test negative for mycoplasma contamination.

Commonly misidentified lines (See ICLAC register)	No misidentified lines used in this study.

Human research participants

Policy information about studies involving human research participants

Population characteristics	Participants were collected from the Taiwan Biobank. Characteristics of participants regarding age, gender and biochemical measures are provided in Table S1.
Recruitment	Participants were recruited from community by Taiwan Biobank.
Ethics oversight	The study was approved by the ethics committee of the institutional review board of Academia Sinica (AS-IRB01-16018)

Note that full information on the approval of the study protocol must also be provided in the manuscript.
